# The Impact of Early Mobilization on the Incidence of Intensive Care Unit-Acquired Weakness in Patients with Sepsis in the Critical Care—The Shinshu Multicenter Prospective Cohort Study (EROSCCS Study)

**DOI:** 10.3390/jcm14165904

**Published:** 2025-08-21

**Authors:** Yasunari Sakai, Kohei Taniuchi, Takuma Karasawa, Ken Matsui, Takeshi Matsumoto, Shota Ikegami, Hiroshi Imamura, Hiroshi Horiuchi

**Affiliations:** 1 Department of Rehabilitation Medicine, Shinshu University Hospital, Nagano 390-8621, Japan; sh.ikegami@gmail.com (S.I.); horiuchih@shinshu-u.ac.jp (H.H.); 2 Department of Rehabilitation Center, Aizawa Hospital, Nagano 390-0814, Japan; tanihei0524@gmail.com; 3 Department of Rehabilitation, Ina Central Hospital, Nagano 396-0033, Japan; jtrh.a17@gmail.com; 4 Department of Rehabilitation, Saku Central Hospital Advanced Care Center, Nagano 385-0051, Japan; neek.no.1122@gmail.com (K.M.); higepapa@ymail.ne.jp (T.M.); 5 Department of Advanced Emergency Critical Care Center, Shinshu University Hospital, Nagano 390-0802, Japan; imamura@shinshu-u.ac.jp

**Keywords:** sepsis patients, early mobilization, intensive care unit-acquired weakness, multicenter observational study

## Abstract

**Background**: Post-Intensive Care Syndrome (PICS), which includes Intensive Care Unit-Acquired Weakness (ICU-AW), can lead to lasting functional impairments even after patients are discharged from the hospital. Early mobilization is a key strategy for preventing ICU-AW, a major contributor to PICS. The primary objective of this study is to assess the impact of early mobilization on ICU-AW in critically ill sepsis patients, while also evaluating the feasibility of a larger, multicenter study through comparison with previous data. **Methods**: This multicenter observational study, conducted in four hospitals in Nagano Prefecture, Japan, from April 2020 to March 2023, included sepsis patients admitted to the ICU or emergency departments. Patients were classified into ICU-AW and non-ICU-AW groups based on admission data. Background factors and discharge outcomes (complications, ADL, physical function) were assessed. Logistic regression analysis was performed to evaluate the relationship between early mobilization and ICU-AW incidence, with a subgroup analysis on the impact of a dedicated team or physiotherapist. **Results**: A total of 154 sepsis patients were enrolled, with 76 (49.4%) diagnosed with ICU-AW at discharge. The most common infection source in ICU-AW patients was the urinary tract (31%). Early mobilization (≥3 days) significantly reduced ICU-AW incidence, with adjusted odds ratios of 3.73 (95% CI = 1.79–7.77) for treatment details and 2.93 (95% CI = 1.22–7.08) for patient factors. However, the presence of a dedicated team or physiotherapist did not significantly affect ICU-AW incidence, with adjusted odds ratios of 0.50 (95% CI = 0.24–10.6) and 0.99 (95% CI = 0.40–2.47), respectively. **Conclusions**: Early mobilization effectively reduced ICU-AW incidence in sepsis patients, though a dedicated team or physiotherapist had no significant impact. Urinary tract infections were the most common infection source in ICU-AW patients. Early mobilization during dialysis for acute kidney injury shows promising potential and warrants further promotion.

## 1. Introduction

Intensive Care Unit-Acquired Weakness (ICU-AW) is a frequent and clinically significant complication among critically ill patients, with reported incidence rates ranging from 25% to 50%, depending on the diagnostic criteria employed and the severity of the underlying critical illness [1,2]. The etiology of ICU-AW is multifactorial, involving factors such as Systemic Inflammatory Response Syndrome (SIRS) [3], the use of neuromuscular blockers [4], corticosteroid administration [5], and prolonged bed rest [6]. ICU-AW has been associated with prolonged mechanical ventilation [7], longer ICU and hospital stays [7,8], and increased mortality [9]. Additionally, it significantly impacts the quality of life of patients following their ICU stay. This is particularly relevant for patients with sepsis and septic shock, a prototypical example of critically ill patients. Sepsis and septic shock cause organ dysfunction and, despite advances in medical care, remain associated with high morbidity and mortality, posing a significant global health burden [10].

In recent years, various therapeutic strategies have been developed for clinical treatment, including electrical stimulation, acupuncture, mobilization therapy, pharmacological interventions, and combination therapies. Although strategies such as the reduction in sedatives and neuromuscular blocking agents, as well as the combined implementation of nutritional therapy and rehabilitation, are considered promising for improving patient outcomes, there is still a lack of studies that have directly evaluated their effects. Among these approaches, early progressive mobilization has been shown to significantly alleviate neuromuscular dysfunction, promote muscle strength recovery, and play a crucial role in the prevention and treatment of ICU-AW [11]. The importance of early mobilization for critically ill patients in the intensive care unit is well recognized in Japan [12]. Additionally, the composition of the care team plays a crucial role in facilitating early mobilization [13]. Regarding the composition of the dedicated team, we have previously reported that early mobilization by a specialized physical therapist or a dedicated team in sepsis patients is effective in reducing acute respiratory complications and improving the rate of activities of daily living (ADL) independence [14,15]. Anekwe also reported in his meta-analysis that early rehabilitation reduces ICU-AW [16], although this study only suggests the potential that early rehabilitation may contribute to the prevention and improvement of ICU-AW; however, the quality and quantity of evidence remain limited. In fact, there is inconsistency in the intervention protocols and definitions across studies, as well as substantial heterogeneity in patient populations, making generalizations difficult and requiring cautious interpretation. Furthermore, it has been shown that Post-Intensive Care Syndrome (PICS), including ICU-AW, can lead to residual functional impairments even after discharge, meaning that recovery requires a prolonged and intensive rehabilitation process [17]. Given these considerations, it is crucial to determine whether early mobilization can effectively prevent the onset of ICU-AW, a major component of PICS, through large-scale multicenter studies. Such studies are essential for improving the medical management of critically ill patients and could significantly advance early mobilization strategies in the future.

This study aims to examine the impact of early mobilization on the incidence of ICU-AW in sepsis patients within a critical care setting. Additionally, the aim is to evaluate the feasibility of this multicenter study.

## 2. Materials and Methods

### 2.1. Study Population

**Figure 1:**
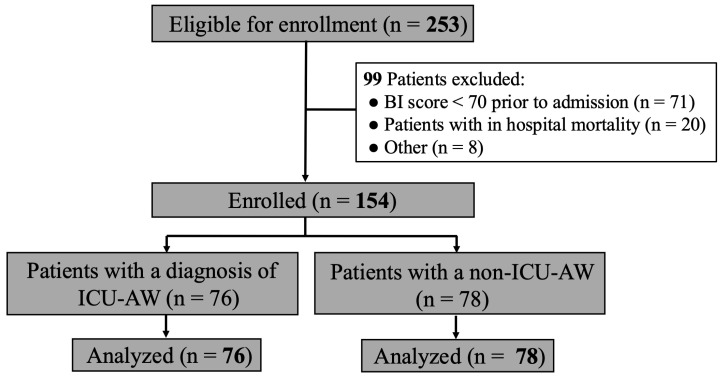
Flow chart of patients included in the study and subject of analysis. Other causes including data missing, head trauma, burns, spinal cord injury, multiple fractures.

This study is a multicenter prospective observational design conducted on sepsis patients admitted to the intensive care unit (ICU) and emergency departments of four acute care hospitals in Nagano Prefecture, Japan, from April 2020 to March 2023. Patients were included if they were 18 years or older, had received intensive treatment, and were diagnosed with sepsis [18] (defined as two or more criteria of systemic inflammatory response plus a proven or strongly suspected infection), sepsis with organ failure, or septic shock (sepsis with hypotension unresponsive to fluid resuscitation). To be enrolled, patients also needed to meet the criteria for baseline functional independence, defined a priori as a barthel index (BI) score ≥ 70, obtained from a proxy describing the patient’s functional status prior to admission [19,20]. The following conditions, which could influence the diagnosis of ICU-AW, were excluded from the analysis: head trauma, burns, spinal cord injury, multiple fractures, septic shock unresponsive to treatment, and patients with in-hospital mortality. A flowchart of patient enrollment is shown in Figure 1.

### 2.2. Ethical Approval

This study was conducted in accordance with the Declaration of Helsinki and was approved by the ethics committee of each hospital (Shinshu University No. 4161). This research was conducted as an observational study, with appropriate safeguards in place to ensure that individual participants could not be identified. Consequently, the requirement for informed consent was formally waived. Instead, we provided an opt-out option. This study was registered with the University Hospital Medical Information Network Clinical Trials Registry, number UMIN000040570 (28 May 2020). Information on the study was published on the bulletin board or webpage according to the discretion of each hospital, allowing patients the opportunity to withdraw participation in the study anytime. To protect the privacy of the participants, all clinical data were anonymized. Moreover, outcome assessors and researchers were blinded to prevent bias.

### 2.3. Methods

All patients received routine therapy and management as per the standard protocols of the ICU and emergency departments, including vital signs monitoring, tube and ventilator care, position management, nutritional support, and other symptomatic and supportive treatments. Early mobilization was integrated into the routine interventions. Early mobilization involved the following components: Patient tolerance and risk were evaluated before each mobilization, and based on the patient’s disease type, consciousness level, and muscle strength, the primary care physician and physical therapist or early mobilization team determined whether to initiate passive mobilization or active exercise. During the bed-leaving phase, active exercises such as standing, sitting, gait training, and walking were performed with support. The frequency and intensity of exercises were adjusted according to the patient’s condition. Additionally, pulmonary rehabilitation (including deep breathing exercises, periodic noninvasive ventilation, and supported coughing), electrical muscle stimulation (EMS) using a General Therapeutic Electrical Stimulator (Homer Ion Co., Tokyo, Japan), and other mobilization techniques were incorporated as needed. If required, a bed with a tilt function (Total Lift Beds; Paramount Bed Co., Tokyo, Japan) was used to facilitate early mobilization. Mobilization sessions were conducted once or twice daily, with each session lasting 20–40 min, until hospital discharge.

Implementation Criteria: Early mobilization was performed in accordance with the discontinuation criteria outlined in the Japanese Clinical Practice Guidelines for Rehabilitation in Critically Ill Patients (2023) [12]. If abnormal vital signs or other high-risk conditions (including persistent elevated intracranial pressure, acute myocardial ischemia, gastrointestinal bleeding, etc.) occurred, the intervention was suspended. The suspended intervention was gradually resumed once the patient’s condition stabilized.

### 2.4. Patient Data Collection During Hospitalization

We investigated each patient’s age, gender, body mass index (BMI), severity score (sequential organ failure assessment score; SOFA score, disseminated intravascular coagulation score; DIC score, procalcitonin; PCT), primary category of infection, treatment details during hospitalization including use of mechanical ventilation (MV) and mobilization implementation information, baseline ADL, discharge outcome measures including complications, patient outcomes, ADL, and physical function. In this study, blinded assessments were conducted on the participants

### 2.5. Medical Research Council (MRC) Score Measurement and ICU-AW Definitions

The MRC score was employed to assess muscle strength and to diagnose ICU-AW at the time of discharge from the ICU or emergency department. The MRC scale comprises 12 items, each rated on a scale from 0 to 5 points, yielding a maximum total score of 60. Higher scores reflect greater muscle strength. A total score of less than 48 points is indicative of ICU-AW [21]. The Barthel Index, which evaluates activities of daily living, consists of 10 items including eating, bathing, dressing, and other self-care activities. The total score ranges from 0 to 100, with higher scores denoting a greater degree of independence in daily functioning [22]. The Short Physical Performance Battery (SPPB) is a comprehensive assessment tool that evaluates physical performance across three domains: balance testing, chair stand testing, and a 4-meter walking speed test. Each domain is scored from 0 to 4 points, with the total score ranging from 0 to 12. Higher scores correspond to better physical performance [23].

### 2.6. Statistical Analysis

Data were tested for normality using the Shapiro–Wilk test. Parametric tests were applied to normally distributed variables, while non-parametric tests were used for non-normally distributed variables. Categorical and continuous data of the characteristics are presented as percentages (%) and median [25%, 75%]. The χ^2^ test was used to compare the categorical variables between ICU-AW participants with and without ICU-AW. The Student’s *t*-test or the Mann–Whitney’s U-test was used to compare continuous variables between two groups. We conducted logistic regression analysis to identify the relationship between early mobilization and the incidence of ICU-AW. Model 1 included early mobilization, catecholamine, corticosteroid, anticoagulant sedative use MV and ICU/Emergency center stay (treatment details model); Model 2 included early mobilization, age, BMI, sex, SOFA score, DIC score, pulmonary complication and delirium complication (patient background severity model). We calculated E values to estimate the potential in effect of unmeasured confounders. Additionally, a supplementary sub-analysis was conducted to investigate the association between the presence or absence of a dedicated team or physiotherapist and the incidence of ICU-AW. In this context, early mobilization was defined using the median number of days to rehabilitation initiation among all study participants—three days—as the cutoff point (3 day≧ vs. 3 day<). The analyses were performed using the STATA version 17 (StataCorp, College Station, TX, USA). Descriptive statistics (mean ± standard deviation or median [25%, 75%]) were calculated. All tests at *p* ≤ 0.05 were considered statistically significant.

## 3. Results

### 3.1. Characteristics of Study Patients

During the study period, a total of 253 sepsis patients were enrolled in the four centers. From those, 99 patients fulfilled the exclusion criteria and 154 met the inclusion criteria. Seventy-six patients (49.4%) were diagnosed with ICU-AW at ICU and emergency center discharged, and 78 patients were not (Figure 1). Baseline characteristics of patients with and without ICU-AW are shown in Table 1. Importantly, no adverse events related to mobilization were observed in this study.

Patients with ICU-AW demonstrated higher use of anticoagulants at baseline (*p* < 0.05).

**Figure 2:**
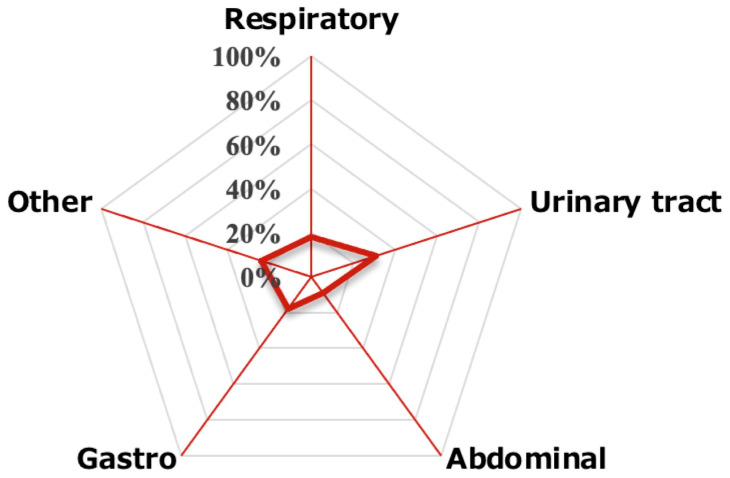
The breakdown of the source of infection in ICU-AW patients. 31% urinary tract, 18% respiratory tract, 18% gastroenteritis, 9% abdominal, 24% other, with urinary tract being the most common.

These patients also had a significantly slower start of mobilization and a lower BI (*p* < 0.05). Finally, the breakdown of the source of infection in ICU-AW patients was 31% urinary tract, 18% respiratory tract, 18% gastroenteritis, 9% abdominal, and 24% other, with urinary tract being the most common (Figure 2).

### 3.2. Outcome Measurement

Outcome measurement of patients with and without ICU-AW are shown in Table 2. Patients with ICU-AW demonstrated lower BI, SPPB total at discharge, and discharge to home rate (*p* < 0.05). These patients also had a significantly higher incidence of pulmonary complications during hospitalization (*p* < 0.05).

### 3.3. The Relationship Between Early Mobilization and the Incidence of ICU-AW

**Figure 3:**
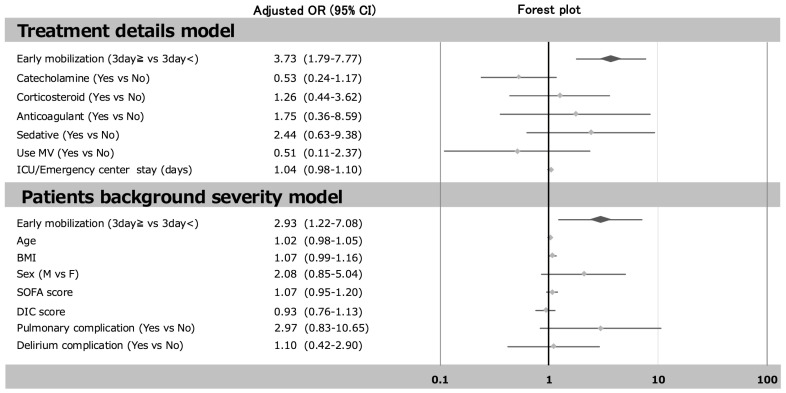
The relationship between early mobilization (3 day≧ vs. 3 day<) and the incidence of ICU-AW. Multivariable analysis indicates the adjusted by early mobilization, catecholamine, corticosteroid, anticoagulant sedative use MV and ICU/Emergency center stay (treatment details model), and early mobilization, age, BMI, sex, SOFA score, DIC score, pulmonary complication, and delirium complication (patient background severity model). Abbreviations: OR, odds ratio; CI, confidence interval.

**Figure 4:**
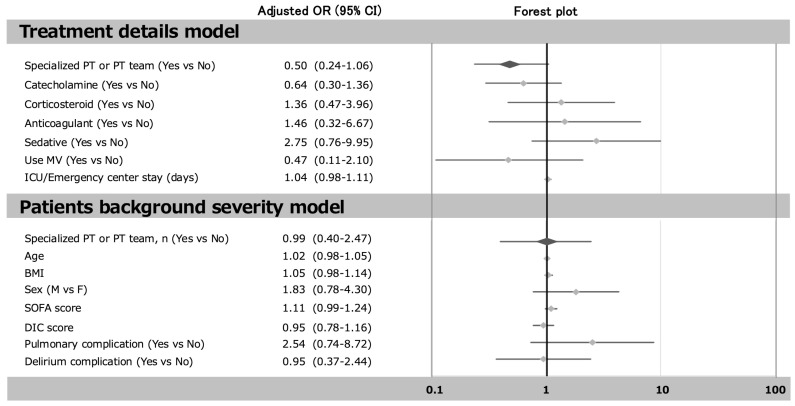
The relationship between presence or absence of a dedicated team or physiotherapist and the incidence of ICU-AW. Multivariable analysis indicates the adjusted by early mobilization, catecholamine, corticosteroid, anticoagulant sedative use MV and ICU/Emergency center stay (treatment details model), and early mobilization, age, BMI, sex, SOFA score, DIC score, pulmonary complication, and delirium complication (patient background severity model). Abbreviations: OR, odds ratio; CI, confidence interval.

Figure 3 and Figure 4 show the logistic regression analysis. After adjustment for catecholamine, corticosteroid, anticoagulant sedative use MV and ICU/Emergency center stay and early mobilization (treatment details model), the odds ratio (OR) early mobilization (3 day≧ vs. 3 day<) for incidence of ICU-AW was 3.73 (1.79–7.77). It was also after adjustment for age, BMI, sex, SOFA score, DIC score, pulmonary complication and delirium complication, and early mobilization (patient background severity model), that the OR early mobilization (3 day≧ vs. 3 day<) for incidence of ICU-AW was 2.93 (1.22–7.08). In contrast, after adjustment for catecholamine, corticosteroid, anticoagulant, sedative use MV and ICU/Emergency department stay, and presence or absence of dedicated team or physiotherapist (treatment details model), the OR presence or absence of dedicated team or physiotherapist for the incidence of ICU-AW was 0.50 (0.24–1.06). It was also after adjustment for age, BMI, sex, SOFA score, DIC score, pulmonary complication and delirium complication, and presence or absence of dedicated team or physiotherapist (patient background severity model), that the OR presence or absence of dedicated team or physiotherapist for incidence of ICU-AW was 0.99 (0.40–2.47).

## 4. Discussion

### 4.1. Key Findings and Strengths

This is a highly valuable multicenter study that demonstrates the effect of early mobilization and the presence or absence of a dedicated team or physiotherapist on the incidence of ICU-AW in sepsis patients within a critical care setting. The study found that early mobilization was effective in reducing the incidence of ICU-AW in sepsis patients. However, the impact of a dedicated team or physiotherapist on the reduction in ICU-AW incidence could not be validated. In addition, patients with ICU-AW were more likely to experience respiratory complications and had poorer physical function outcomes at discharge, leading to a lower rate of discharge to home. The early mobilization protocol used in this multicenter study was found to be highly feasible and adaptable for implementation in a general intensive care setting.

### 4.2. Early Mobilization and the Role of a Dedicated Team or Physiotherapist

The incidence of ICU-AW in our study was higher than that reported in previous studies [5,6], which may reflect the inclusion of a cohort with greater severity of illness. Patients with ICU-AW had a higher baseline use of anticoagulants (*p* < 0.05). These patients also experienced a significantly delayed initiation of mobilization and had lower BI scores (*p* < 0.05). These findings suggest that the intensive care required for the various complications of sepsis may serve as a barrier to early mobilization, and that, by the time mobilization began, these patients had already experienced a reduction in their ADL, making them more susceptible to developing ICU-AW. In fact, it has been reported that patients with ICU-AW experience a significant decrease in muscle mass between days 1 and 5 of intensive care, indicating that rapid muscle loss occurs immediately after ICU admission [24].

The primary outcome of the study was that the ICU-AW group had more complications during hospitalization, poorer physical function and ADL at discharge, and a lower rate of return to home compared to the non-ICU-AW group. On the other hand, patients in the non-ICU-AW group who underwent early mobilization showed improved self-care compared to those with ICU-AW. Mobilization treatments such as turning, massage, and passive flexion and extension of the limb joints effectively stimulated the joint extension-contraction cycle and helped maintain muscle flexibility. ICU-AW, a hallmark manifestation of PICS, is known to develop early in the course of critical illness, primarily due to accelerated protein catabolism associated with severe conditions such as sepsis [25]. These conditions are closely associated with systemic inflammatory responses. Conversely, findings from basic research using septic mice models have demonstrated that exercise therapy can attenuate the progression of sepsis-induced inflammation [26].

Moreover, it has been reported that exercise intensity plays a crucial role in the recovery of physical function in ICU-AW patients [27]. Therefore, it is desirable to provide higher-intensity exercise therapy to ICU-AW patients; however, various barriers to early mobilization in clinical practice often prevent its implementation [28]. The results of this study did not allow us to verify the impact of the presence or absence of a specialized team or physiotherapist on the reduction in ICU-AW incidence. However, a single-center study we led suggests that specialist physiotherapists treating sepsis patients in the intensive care unit can raise awareness of rehabilitation, increase requests for rehabilitation, and improve team collaboration in the care of these patients [14,15]. Although early mobilization is becoming increasingly widespread in clinical practice, the methods, duration, and intensity of mobilization vary between centers. We believe this variation reflects differences in the content of mobilization protocols. Positive results were obtained in a single-center study led by our team, and based on these findings, we consider it crucial to reduce the disparities in mobilization content and intensity between centers in the future. Moreover, it may be essential to have dedicated physiotherapists who are capable of overcoming the barriers to mobilization and possess the necessary knowledge and risk management skills to deliver more effective exercise therapy.

### 4.3. ICU-AW and Acute Kidney Injury (AKI)

The most common source of infection in ICU-AW patients was the urinary tract, accounting for 31%. Among ICU-AW patients with urinary tract infections, those with AKI were more likely to require hemodialysis during their clinical course. The incidence of ICU-AW is high in patients with AKI [29], highlighting the importance of early rehabilitation. In the EROSCCS study we led, approximately 36% of sepsis patients in the ICU also had AKI; however, there was no significant difference in the incidence of ICU-AW between patients with AKI and those without. These findings suggest that early mobilization can be effective in reducing ICU-AW even in patients with AKI. However, in patients with AKI, factors such as unstable hemodynamics, slow fluid removal, orthostatic hypotension due to antigravity shifts, and complications with vascular access (e.g., bleeding, poor blood flow, and accidental removal) are likely to hinder early mobilization. Therefore, it is essential to develop and adapt mobilization strategies to better promote early mobilization in patients with AKI complications in the future.

Finally, given the significant relationship between ICU-AW and PICS, the onset of ICU-AW not only increases complications but also necessitates long-term rehabilitation. This underscores the critical need for early mobilization, with a particular emphasis on preventing the development of ICU-AW. Although early mobilization is feasible across various clinical settings, we believe that the establishment of a dedicated system will further enhance its effectiveness in the future.

There are several potential limitations of this study. First, while this is a multi-center study, its observational design limits the ability to establish a causal relationship between early mobilization and the reduction in the incidence of ICU-AW. Furthermore, participant self-selection into the cohort introduces the possibility of selection bias. Individuals and families with higher health consciousness or differing baseline characteristics may have been overrepresented, which could impact the internal validity and generalizability of the findings. Second, cognitive and emotional status were not assessed, which may limit the interpretation of the effects. Additionally, the baseline characteristics of the two groups did not match, potentially introducing bias. These limitations could be addressed by adjusting for background factors, such as through propensity score matching or by conducting multicenter randomized controlled trials. Third, the generalizability of the findings is limited, as the study was conducted in secondary and tertiary emergency centers with a large proportion of severely ill patients. Finally, more detailed data on clinical indications are required, as this study focused solely on the timing of early mobilization and lacked information regarding its content, duration, and intensity.

## 5. Conclusions

Early mobilization was effective in reducing the incidence of ICU-AW in sepsis patients in critical care settings. However, the impact of having a dedicated team or physiotherapist on the reduction in ICU-AW incidence could not be confirmed. Additionally, the most common source of infection in ICU-AW patients was the urinary tract, and early mobilization during dialysis for AKI shows promising potential and should be further encouraged.

## Figures and Tables

**Table 1:** Baseline characteristics of patients with ICU-AW and without.

Variables	ICU-AW (*n* = 76)	Non-ICU-AW (*n* = 78)	*p*-Value
Age (years)	76.8 ± 12.9	74.5 ± 11.6	0.25
Men/women, *n* (%)	44 (58)/32 (42)	54 (69)/24 (31)	0.144
BMI (kg/m^2^)	21.4 (19.4, 23.8)	21.8 (19.9, 24.8)	0.255
**Severity score**			
SOFA score	7 (5, 12)	6 (4, 9)	0.074
DIC score	2 (1, 4.5)	2 (1, 4)	1.000
PCT (ng/mL)	31.0 (2.27, 55.2)	22.3 (2.8, 97.9)	0.721
**Primary category of infection**			
Respiratory, *n* (%)	14 (18)	12 (15)	
Urinary tract, *n* (%)	23 (31)	31 (40)	
Abdominal, *n* (%)	7 (9)	10 (13)	0.22
Gastroenteritis, *n* (%)	15 (18)	16 (21)	
Others, *n* (%)	17 (24)	9 (11)	
**Treatment details**			
Catecholamine, *n* (%)	47 (62)	54 (69)	0.334
Corticosteroid, *n* (%)	14 (18)	11 (14)	0.468
Anticoagulant, *n* (%)	6 (8)	4 (5)	0.016
Sedative, *n* (%)	20 (26)	15 (19)	0.145
Use MV, *n* (%)	14 (18)	12 (15)	0.615
Start of mobilization (day)	4 (3, 4)	2 (1, 4)	*p* < 0.001
Specialized PT or PT team, *n* (%)	43 (57)	51 (65)	0.263
ICU/Emergency center stay (days)	5 (2, 9)	4 (2.3, 6)	0.168
**Baseline ADL**			
Barthel index baseline (points)	0 (0, 20)	10 (0, 35)	0.015

Definition of abbreviations: ICU, intensive care unit; BMI, body mass index; SOFA, sequential organ failure assessment; DIC, disseminated intravascular coagulation; PCT, procalcitonin; MV, mechanical ventilation; PT, physical therapist; ADL, activities of daily living. Start of mobilization (day), a lower number reflects an earlier start of mobilization, representing a shorter interval between admission and the initiation of mobilization efforts. Values are mean (standard deviation), median (interquartile range), or *n* (%).

**Table 2:** Outcome measurement of patients with and without ICU-AW.

Variables	ICU-AW (*n* = 76)	Non-ICU-AW (*n* = 78)	*p*-Value
**Outcome**			
Barthel index discharge (points)	65 (17.5, 87.5)	90 (80, 100)	*p* < 0.001
SPPB total (points)	6.5 (2, 11.5)	10 (5, 12)	0.042
Delirium complication, *n* (%)	19 (25)	20 (26)	0.864
Plumonary complication, *n* (%)	18 (24)	5 (6)	0.002
Dischage to home, *n* (%)	29 (38)	62 (79)	0.005

Definition of abbreviations: ICU, intensive care unit; SPPB, short physical performance battery. Values are mean (standard deviation), median (interquartile range), or *n* (%).

## Data Availability

The data that support the findings of this study are available from the corresponding author [Y.S.], upon reasonable request.

## Abbreviations

PICS	Post-Intensive Care Syndrome
ICU-AW	Intensive Care Unit-Acquired Weakness
ICU	Intensive Care Unit
ADL	Activities of Daily Living
SIRS	Systemic Inflammatory Response Syndrome
BI	Barthel Index
EMS	Electrical Muscle Stimulation
BMI	Body Mass Index
SOFA	Sequential Organ Failure Assessment
DIC	Disseminated Intravascular Coagulation
PLT	Procalcitonin
MV	Mechanical Ventilation
MRC	Medical Research Council
SPPB	Short Physical Performance Battery
AKI	Acute Kidney Injury
